# MBR-UV/Cl_2_ system in treating polluted surface water with typical PPCP contamination

**DOI:** 10.1038/s41598-020-65845-w

**Published:** 2020-06-01

**Authors:** Dan Liu, Kang Song, Guojun Xie, Lu Li

**Affiliations:** 1 School of the Environment and Safety Engineering, Jiangsu University, Zhenjiang, 212013 China; 2 State Key Laboratory of Freshwater Ecology and Biotechnology, Institute of Hydrobiology, Chinese Academy of Sciences, Wuhan, 430072 China; 3 State Key Laboratory of Urban Water Resource and Environment, Harbin Institute of Technology, Harbin, 150090 China

**Keywords:** Environmental sciences, Engineering

## Abstract

This study proposed the membrane bioreactor–ultraviolet/chlorine (MBR-UV/Cl_2_) process for treating polluted surface water with pharmaceutical personal care product (PPCP) contamination. Results showed that MBR-UV/Cl_2_ effectively removed the organic matters and ammonia at approximately 80% and 95%. MBR-UV/Cl_2_ was used in the removal of sulfadiazine(SDZ), sulfamethoxazole(SMZ), tetracycline(TC), oxytetracycline(OTC), ciprofloxacin(CIP), ofloxacin(OFX), erythromycin(ERY), roxithromycin(ROX), ibuprofen(IBU) and, naproxen(NAX) at 12.18%, 95.61%, 50.50%, 52.97%, 33.56%, 47.71%, 87.57%, 93.38%, 93.80%, and 71.46% in which their UV/Cl_2_ contribution was 12.18%, 95.61%, 29.04%, 38.14%, 25.94%, 7.20%, 80.28%, 33.79%, 73.08%, and 23.05%, respectively. The removal of 10 typical PPCPs using UV/Cl_2_ obtained higher contributions than those of the MBR process, except OTC, ROX, and IBU. The UV/Cl_2_ process with 3-min hydraulic retention time and chlorine concentration at 3 mg/L effectively removed the trace of PPCPs. MBR-UV/Cl_2_ has the potential to be developed as an effective technology in treating polluted surface water with PPCP contamination.

## Introduction

Freshwater lakes are important drinking water sources facing pharmaceutical personal care product (PPCP) contamination^1–3^. Yan *et al*.^1^ investigated five groups of antibiotics in the surface water of Yangtze estuary over four seasons, the total concentration of the 5 classes of antibiotics detected were 100–300 ng/L. PPCPs used in medicine, animal husbandry, and aquaculture were unintentionally discharged into the lakes without proper treatment. The PPCP residence time in lakes could increase and cause other problems due to the slow circulation in this body of water^4,5^. The PPCP residues in lakes can cause serious problems to the treatment facilities of drinking water because the treatment process of conventional drinking water is not designed for treating polluted water with this type of contamination^6–8^. Li *et al*.^6^ investigated the performance of removing PPCPs using two different conventional treatment techniques and revealed that the existing treatment process of drinking water should be improved to increase the eliminating efficiency of emerging contaminants and ensure proper water quality. The antibiotics and improper treatment processes used in the treatment plants of drinking water could enhance the antibiotic-resistant genes^9,10^. Hence, developing an effective alternative technology in treating drinking water, specifically these emerging contaminants, is necessary.

Apart from the increasing contamination of emerging trace PPCPs, surface water quality is also deteriorating and arose in the process of treating polluted surface water. The organic matters and ammonia concentration in polluted surface water were relatively higher than those of common-source water^11,12^. Li and Chu^13^ reported that a membrane bioreactor (MBR) could potentially treat polluted surface water for drinking water supply. Li *et al*.^14,15^ found that an attached growth MBR (aMBR) is effective in treating polluted surface water without adding any sludge, wherein the carrier polyvinyl alcohol gel (PVA-gel) was the main contributor for water purification. The aMBR system has shown a potassium permanganate index (COD_Mn_) removal efficiency of two times higher than the system of direct membrane filtration. The biological process provided by the carrier PVA-gel was the main contributor for the removal of organic matter in aMBR. The recalcitrant organic matters were rarely removed by this system and required other processes. Polluted surface water always faces high contaminations, including PPCPs, compared with lake water. The water demand by human beings increases, and the water quality worldwide deteriorates; thus, the removal of PPCPs from polluted surface water should be taken into account.

Li *et al*.^16^ reported that the integration of an advanced oxidation process into the MBR system remarkably improved the removal of recalcitrant organic matters. The COD_Mn_ and UV_254_ values (a simple index stand for recalcitrant organic matters) were reduced with the increment in the recirculation ratio. Yang *et al*.^17^ reviewed the occurrence and removal of PPCPs in the treatment plant of drinking water and found that advanced treatment technologies effectively treat contaminated water with PPCPs although a large variation existed in PPCP removal between the drinking water and wastewater treatment processes. The compound characteristics and process-specific factors were related to the PPCP removal in the treatment process. Yang *et al*.^18^ implemented the ultraviolet/chlorine (UV/Cl_2_) water purification process for the degradation of commonly found PPCPs. Their results showed that UV/Cl_2_ has enhanced the removal of PPCPs, which is attributed to the weaker effect of hydroxyl and chlorine radicals and UV/Cl_2_ together with postchlorination in the disinfection by-product (DBP) formation enhancement compared with the UV/H_2_O_2_ process^19–21^. Gao *et al*.^22^ investigated the kinetics and mechanism of naproxen (NAX) removal through the UV/chlorine process and reported that UV/chlorine is a promising technology for treating water polluted with emerging contaminants. The NAX degradation in this process was associated with decarboxylation, demethylation, and hydroxylation. The UV/Cl_2_ process could generate additional DBP with the high ammonia concentration in the feedwater. Thus, in treating polluted surface water, which has higher ammonia than common-source water, a suitable pretreatment is necessary to remove ammonia remarkably.

This study investigated the removal of 10 typical PPCPs from polluted surface water by using MBR combined with the UV/Cl_2_ process. The performance of PPCPs removal from polluted surface water through the MBR-UV/Cl_2_ system was investigated. The contribution of PPCP removal through MBR and the UV/Cl_2_ process was analyzed. The performance of different PPCP removal from polluted surface water was investigated to provide information in the treatment sector of drinking water.

## Materials and Methods

### Membrane bioreactor system setup

**Figure 1:**
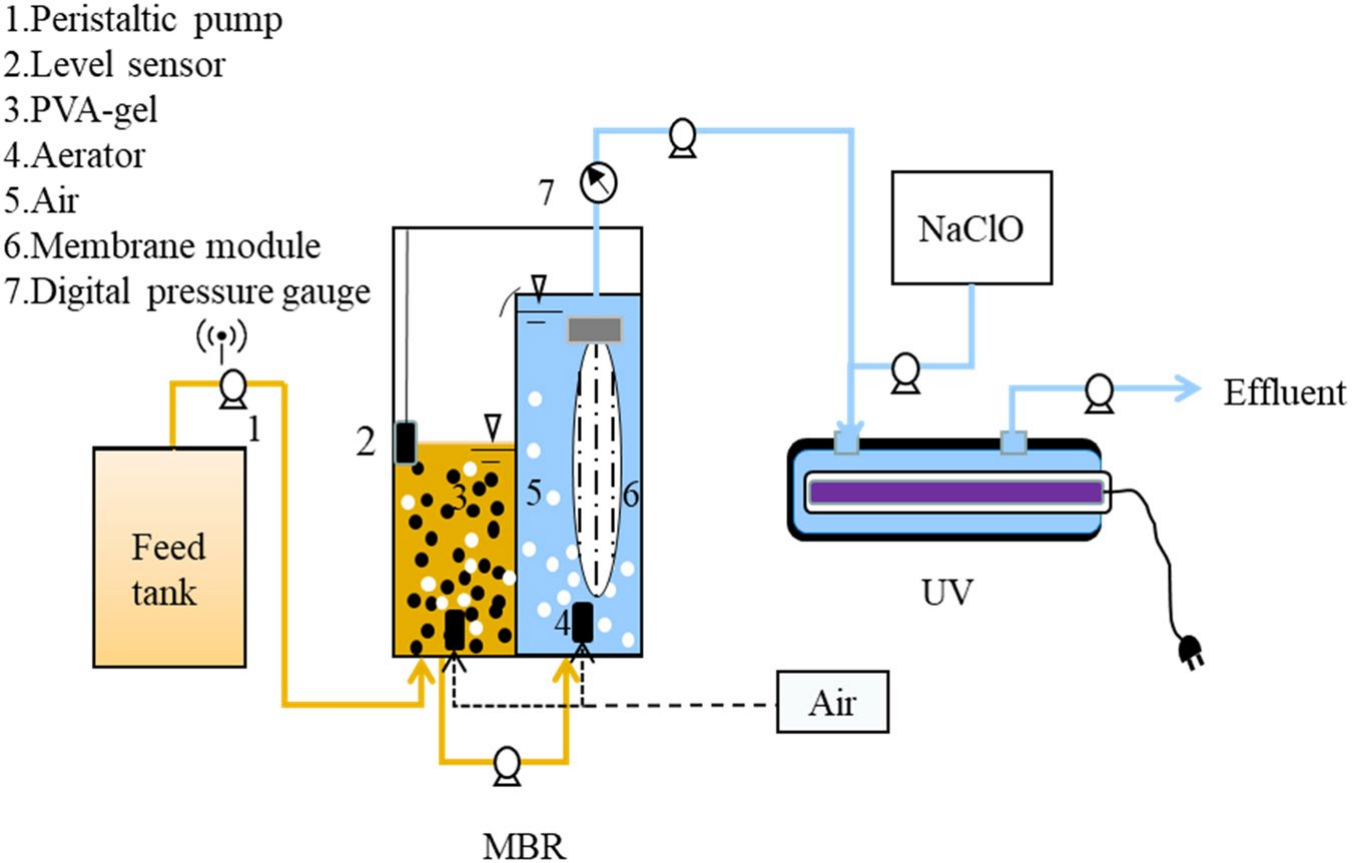
The schematic of MBR-UV/Cl_2_ system for treating polluted surface water.

Figure 1 shows the MBR-UV/Cl_2_ system setup. The membrane used had a pore size and surface area of 0.1 μm and 0.1 m^2^, respectively (Sumitomo, Japan). The UV/Cl_2_ reactor was prepared using a commercial stainless-steel UV sterilizer with the UV lamp (8 W, 254 nm) tube located at the center and the water surrounding the UV lamp tube inside the reactor. The MBR-UV/Cl_2_ system was first setup and running for approximately 2 months in the laboratory, and then, typical PPCPs were added in the feedwater. The feedwater was first sent to the biocarrier side for biodegradation using the PVA-gel (Kuraray, Japan), and then, the water was filtrated in the membrane module. The PVA-gel was immobilized by conventional wastewater treatment plants activated sludge for 2 weeks, and then gently washed with MilliQ water before transferring to the system to avoid the introduction of activated sludge. The membrane permeate was pumped out at a flow rate of 2 L/h. The PVA-gel filling ratio and hydraulic retention time (HRT) were 5% and 2.5 h, respectively, and the sludge retention time in this study was almost infinity. The UV/Cl_2_ reactor was operated with HRT of 3 min and a chlorine concentration of 3 mg/L, which is equal to 0.04 mM. Chlorine was prepared with NaClO. The MBR permeate and NaClO were sent to the UV/Cl_2_ reactor together for reaction.

### Character of the feedwater

Synthetic polluted surface water with COD_Mn_ approximately 10 mg/L and NH_4_^–^N of roughly 3 mg/L was used as feed water in this study. The carbon source was prepared with glucose (C_6_H_12_O_6_), and the nitrogen source was prepared with NH_4_Cl. Tap water was used for dilution to provide a suitable amount of trace elements. The following were the basic physical-chemical parameters of feedwater: dissolved oxygen (DO) 1.7 ± 2.0 mg/L, temperature 26.3 °C ± 1.2 °C, and pH 7.0 ± 0.2; aMBR effluent: DO 7.5 ± 0.2 mg/L, temperature 26.3 °C ± 0.8 °C, and pH 7.7 ± 0.1; aMBR-UV/Cl_2_ effluent: DO 7.5 ± 0.2 mg/L, temperature 25.7 °C ± 0.4 °C, and pH 8.0 ± 0.1.

### Basic water quality parameters

Water quality was analyzed using the following standard methods^23^. COD_Mn_ was used as an indicator for organic matters in the analysis of polluted surface water, and ammonia (NH_4_^–^N) was tested through Nessler’s method.

### PPCP pretreatment

The water samples were filtered using a 0.45-μm glass fiber membrane (Millipore, USA) with a sample volume of 200 mL each. PPCPs in the water samples were concentrated through solid-phase extraction (SPE) with Oasis HLB cartridges (6 mL, 200 mg, Waters, USA). The detailed SPE process was referred to our previous publication^3^.

### PPCP addition and detection

The 10 PPCP standards, namely, sulfadiazine (SDZ), sulfamethoxazole (SMZ), tetracycline (TC), oxytetracycline (OTC), ciprofloxacin (CIP), ofloxacin (OFX), erythromycin (ERY), roxithromycin (ROX), ibuprofen (IBU), and NAX, were purchased from Solar-bio (China). Each PPCP was added into the feedwater at 200 ng/L. PPCPs were first dissolved with methanol and then added to the feedwater. The feedwater and PPCPs were prepared daily and mixed thoroughly in the feed tank. PPCPs were detected using the Waters ACQUITY UPLC H-class-Xevo TQ MS triple quadrupole MS/MS spectrometer equipped with an electrospray ionization source (Waters, USA). The detailed detection process was referred to our previous publication^3^.

## Results and Discussion

### Removal performance of COD_Mn_ and NH_4_^–^N

**Figure 2:**
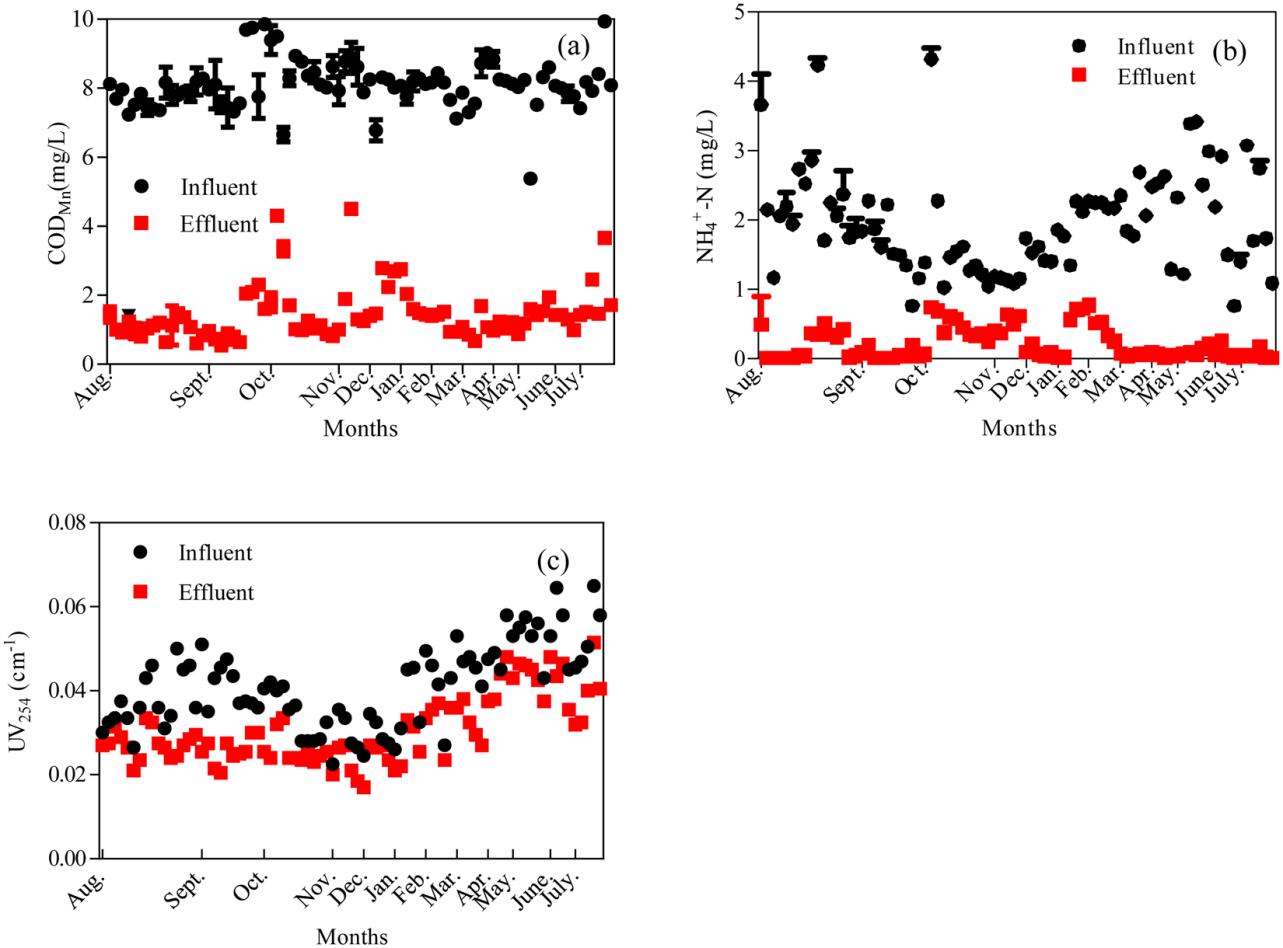
The removal performance of (**a**) COD_Mn_, (**b**) NH_4_-N and (**c**) UV_254_ by membrane bioreactor system.

**Figure 3:**
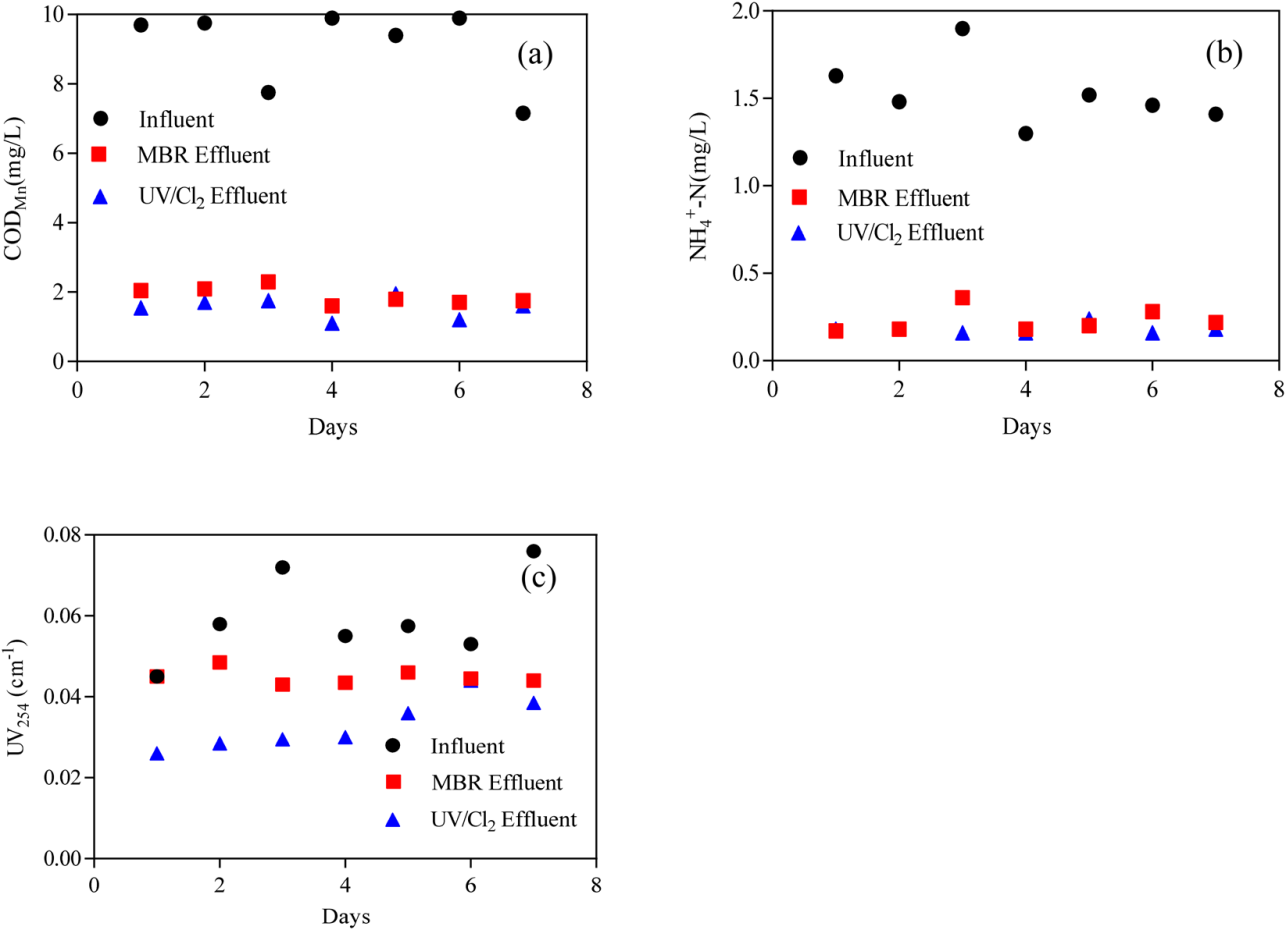
The removal performance of (**a**) COD_Mn_, (**b**) NH_4_-N and (**c**) UV_254_ by UV/Cl_2_ process.

Figure 2 shows the removal of COD_Mn_, NH_4_^–^N, and UV_254_ via the aMBR system. The removal of COD_Mn_, NH_4_^–^N, and UV_254_ were approximately 80%, 95%, and 20%, respectively. The aMBR system has shown good performance in treating polluted surface water with COD_Mn_ and NH_4_^–^N but ineffective in treating those with recalcitrant organic matters. After operating for 2 months, the 10 typical PPCPs were added into the MBR system. Figure 2 shows that the removal of COD_Mn_, NH_4_^–^N, and UV_254_ in PPCPs through the MBR system was unaffected. This finding suggested the in evident inhibition of the biological process for organic matter and ammonia removal with the presence of trace amounts of PPCPs in the feedwater. This finding also implied that with the presence of PPCPs in polluted surface water, biological process, such as MBR, could be used for water purification. Figure 3 depicts the UV/Cl_2_ performance in treating the MBR effluent. UV/Cl_2_ improved the COD_Mn_, NH_4_^–^N, and UV_254_ removal, specifically for UV_254_. The UV/Cl_2_ process contributed to over 50% of the UV_254_ removal. This finding implied that the advanced oxidation process of UV/Cl_2_ had a strong effect on the removal of recalcitrant organic compounds. This process could also be expected to have effects on the removal of PPCPs from this system^24^. The UV/Cl_2_ process could format the hydroxyl radicals (·OH) and other reactive chlorine species, which could be effective in treating the organic matters in the system^25^. The predominant free chlorine species in aqueous solutions are aqueous dechlorane (Cl_2_), hypochlorous acid (HOCl), and hypochlorite ion (ClO^−^), which absorb UV light at the wavelength range of 200 − 375 nm. Some organic matters resistant to molecular chlorine oxidation and UV photolysis could be degraded through the UV/Cl_2_ process. The photolysis of HOCl or ClO^−^ via UV could generate high reactive radicals (·OH and Cl·) and thus promote the removal of recalcitrant organic matter^26^.

### PPCP removal performance through the MBR-UV/Cl_2_ system

**Figure 4:**
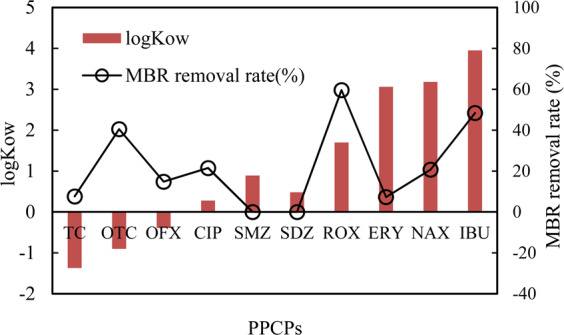
Typical antibiotics removal by membrane bioreactor system.

Figure 4 shows the removal of 10 typical PPCPs via MBR. The MBR system clearly shows the poor removal of PPCPs. The PPCP removal in MBR was 7.62%, 40.51%, 14.83%, 21.49%, 0%, 0%, 59.59%, 7.29%, 20.72%, and 48.41% for TC, OTC, OFX, CIP, SMZ, SDZ, ROX, ERY, NAX, and IBU, respectively. This finding suggested that the biological process and membrane rejection demonstrated a low contribution to PPCP removal. The MBR system was ineffective in treating sulfonamides. The removal of OTC, ROX, and IBU was relatively higher than that of other PPCPs. Azimi *et al*.^27^ reported that SMZ from synthetic wastewater with a concentration range of 5 − 120 mg/L could be removed in the system of a rotating biological contactor with HRT from 12 h to 72 h. This finding suggested that SMZ degradation through the biological process required high HRT. HRT is always short in treating the polluted surface water, specifically the treatment of drinking water. Thus, the removal of PPCPs in the drinking water sector always requires the assistance of advanced oxidation processes.

The molecular weights and octanol–water partition coefficients (log Kow) of the investigated PPCPs ranged from −1.37 (TC) to 3.95 (IBU), as shown in Table 1^28,29^. The membrane surface adsorption related to the log Kow value could be the main reason for the removal of micropollutants. PPCPs demonstrating low lipophilicity, high hydrophilicity, and low log Kow implied their inability for adsorption on the membrane surface. PPCPs with high log Kow (over 4.5) indicated their tendency for adsorption on the membrane surface^30^. This finding was consistent with the results of this study, wherein the high log Kow value obtains high removal in the MBR system. ERY has a high log Kow value but low removal through MBR. This finding was likely due to the reduction of hydrophobicity in PPCPs after deprotonation^31^. TC, OTC, OFX, and CIP obtained low log Kow values, and their removal performance in MBR was not the minimum. The removal of these PPCPs could be attributed to biodegradation and membrane rejection.

**Table 1:** Chemical structure and Log Kow values of ten typical antibiotics used.

Product Name	Molecular Weight (g/mol)	Structural Formula	logKow
Sulfadiazine (SD)	250.28		0.48
Sulfamethoxazole (SMZ)	253.27		0.89
Tetracycline (TC)	444.44		−1.37
OxyTetracycline (OTC)	460.44		−0.9
Ciprofloxacin (CIP)	331.35		0.28
Ofloxacin (OFX)	361.37		−0.39
Erythromycin (ERY)	733.94		3.06
Roxithromycin (ROX)	837.05		1.7
Ibuprofen (IBU)	206.28		3.95
Naproxen (NAX)	230.26		3.18

### Analysis of PPCP removal contribution in the MBR-UV/Cl_2_ system

**Figure 5:**
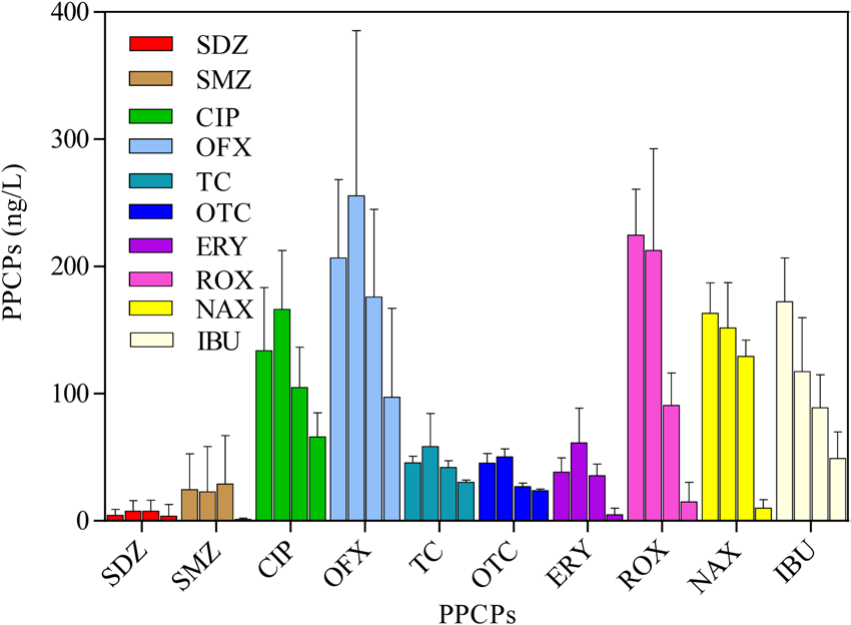
Typical antibiotics removal by MBR-UV/Cl_2_ system.

Figure 5 shows the removal of PPCPs through each process of the MBR-UV/Cl_2_ system. The PPCPs detected from the feedwater and the entire system was remarkably different in each PPCP. The concentration of CIP, VFX, ROX, NAX, and IBU was approximately 200 ng/L in the feedwater, whereas that of SDZ, SMZ, TC, OTC, and ERY was low even in the feed water. This finding could be attributed to the low recovery rate. As shown in Fig. 5, the increased PPCP concentration after the biocarrier treatment (SDZ, CIP, VFX, TC, OTC, and ERY) could be attributed to error and the accumulation of PPCPs retained in the PVA-gel in the carrier side of MBR. ROX, NAX, and IBU with high log Kow values exhibited good removal performance in the carrier side. These PPCPs could likely be adsorbed on the surface of the PVA-gel and thus improve its retention time in the carrier side; hence, the bioprocess and adsorption contributed to the removal of these PPCPs^32,33^. Membrane rejection showed a minimal effect on antibiotics SDZ and SMZ, as shown in Fig. 5. The other PPCPs, including CIP, VFX, TC, OTC, ERY, ROX, NAX, and IBU, showed good rejection with the membrane with a rejection rate of 21.50%, 14.83%, 7.62%, 40.51%, 7.29%, 54.26%, 13.73%, and 16.49%, respectively. Membrane rejection for these typical PPCPs was remarkably higher than the contribution of PVA-gel. Table 2 also shows that the main contribution of PPCP removal through the MBR system was attributed to the membrane process and the carrier side of PVA-gel biodegradation. This finding suggested that the PPCP removal for the treatment of polluted surface water in the MBR system was mainly attributed to membrane rejection and the bioprocess^29,34,35^.

**Table 2:** The average removal efficiency of each PPCPs in difference processes of the MBR-UV/Cl_2_ system.

Processes	SDZ	SMZ	CIP	OFX	TC	OTC	ERY	ROX	NAX	IBU
Carrier side	−78.24%	6.89%	−24.38%	−23.73%	−28.50%	−10.81%	−59.68%	5.33%	6.99%	31.92%
MBR	−76.37%	−17.54%	21.50%	14.83%	7.62%	40.51%	7.29%	59.59%	20.72%	48.41%
MBR-UV/Cl_2_	12.18%	95.61%	50.54%	52.97%	33.57%	47.71%	87.57%	93.38%	93.80%	71.46%
Membrane	1.88%	−24.43%	45.88%	38.55%	36.13%	51.32%	66.97%	54.26%	13.73%	16.49%
UV/Cl_2_	88.55%	100.00%	29.04%	38.14%	25.94%	7.20%	80.28%	33.79%	73.08%	23.05%

**Figure 6:**
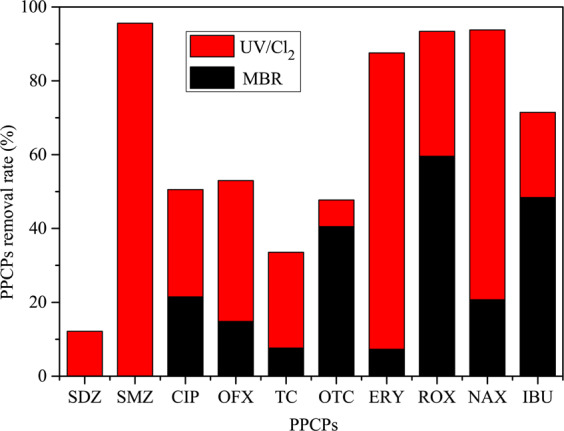
Antibiotics removal contribution by MBR and UV/Cl_2_.

Figure 6 and Table 2 show the contribution of MBR and UV/Cl_2_ to the PPCP removal. The MBR-UV/Cl_2_ system removed 12.18%, 95.61%, 50.50%, 52.97%, 33.56%, 47.71%, 87.57%, 93.38%, 93.80%, and 71.46% of SDZ, SMZ, CIP, OFX, TC, OTC, ERY, ROX, NAX, and IBU, respectively. Moreover, the UV/Cl_2_ contribution to SDZ, SMZ, CIP, OFX, TC, OTC, ERY, ROX, NAX, and IBU removal was 99.55%, 113.15%, 29.04%, 38.14%, 25.94%, 7.20%, 80.28%, 33.79%, 73.08%, and 23.05%, respectively. The PPCPs SDZ, CIP, OFX, TC, OTC, and ERY accumulated in the biological process. The membrane showed low rejection for SDZ and SMZ. The UV/Cl_2_ process demonstrated a higher contribution than the MBR process, except for OTC, ROX, and IBU. This finding suggested that the UV/Cl_2_ process with 3-min HRT and 3 mg/L chlorine concentration could effectively remove the trace of PPCPs from polluted surface water. The removal of sulfonamide antibiotics through the UV/Cl_2_ process could be attributed to the bond-breaking reactions occurring between −SO_2_ − and the side atoms, and the C–S and N–H bonds^28^. The PPCPs removal through the UV/Cl_2_ process could also be attributed to the synergistic effect by a generation of hydroxyl radicals and reactive chlorine species^36,37^.

### Research perspective

This study revealed that the MBR-UV/Cl_2_ process was effective in treating the polluted surface water with PPCP contamination. The MBR system mainly contributed to the removal of organic matter and ammonia, whereas the UV/Cl_2_ process was instrumental in the PPCP removal. The pre-MBR process effectively removed organic matters and ammonia and reduced the turbidity of the water. This finding remarkably reduced the negative effects of UV irradiation and mitigated the consumption of chlorine. The post-UV/Cl_2_ process could focus on the removal of PPCPs, which was not removed by the MBR process. The accumulation of antibiotic-resistant genes and changes in the system should be considered because the biological process could potentially enhance this build-up^38–40^. Under the condition of treating polluted surface water through the established system, the potential of DBP formation from the effluent should also be compared in future studies^41–43^. Hence, additional information could be provided in the development of MBR-UV/Cl_2_ for treating polluted surface water.

## Conclusion

This study developed an advanced oxidation process of combining MBR and UV/Cl_2_ for treating polluted surface water with PPCPs contamination. The MBR-UV/Cl_2_ system demonstrated good performance in polluted surface water treatment and PPCP removal. The removal of COD_Mn_ and ammonia was highly based on the contribution of MBR. The PPCP removal was attributed to the UV/Cl_2_ process. Membrane rejection showed a high contribution to PPCP removal, whereas the bioprocess in MBR exhibited low removal performance in PPCPs. The existence of PPCPs failed to affect the removal of organic matter and ammonia in polluted surface water. This finding implied that MBR-UV/Cl_2_ has the potential to be developed as an effective technology in treating polluted surface water with PPCP contamination.
